# Psychometric properties, validity and insights of the School Bullying Questionnaire (CIE-A) in secondary schools of the Valencian Community (Spain)

**DOI:** 10.1371/journal.pone.0259392

**Published:** 2021-11-08

**Authors:** Sergio A. Useche, Eliseo Valle, Raquel Valle-Escolano, Natura Colomer-Pérez

**Affiliations:** 1 Faculty of Psychology, University of Valencia, Valencia, Spain; 2 Department of Education and School Management, University of Valencia, Valencia, Spain; 3 School of Law and Social Sciences, University Carlos III of Madrid, Getafe, Spain; 4 Department of Nursing, Faculty of Nursing and Chiropody, University of Valencia, Valencia, Spain; Aalborg University, DENMARK

## Abstract

Besides its several threats to health, welfare, social and academic development and performance of kids and teenagers, school bullying remains highlighted as one of the most relevant related challenges for educational, behavioral and legal sciences worldwide. Moreover, the lack of research on the field and the crucial but unattended need to count on psychometrically suitable and valid tools to detect school bullying make difficult understanding its contexts, dynamics and possible solutions. **Objective** The aim of this study was to thoroughly present in detail the psychometric properties and validity issues of the School Bullying Questionnaire (CIE-A) among secondary students. **Methods** A regionwide sample of 810 (47.2% girls) secondary students attending to 21 schools across the Valencian Community (Spain), aged M *=* 14.40 (SD = 1.61) years, responded to a paper-based questionnaire containing the 36-item version of the CIE-A and various scales related to psychosocial health and wellbeing, used as criterion variables. **Results** The outcomes of this study suggest that the CIE-A has a clear factor structure, an optimal set of item loadings and goodness-of-fit indexes. Further, that CIE-A has shown good internal consistency and reliability indexes, coherent associations with other mental health and academic performance variables, and the possibility to assess gender differences on bullying-related factors among secondary students. **Conclusion** The CIE-A may represent a suitable tool for assessing bullying in a three-factorial approach (*i*.*e*., victimization, symptomatology, and intimidation), offering optimal psychometric properties, validity and reliability insights, and the potentiality of being applied in the school environment. Actions aimed at improving the school coexistence and the well-being of secondary students, targeting potential bullied/bully profiles or seeking to assess demographic and psychosocial correlates of bullying among teenagers, might get benefited from this questionnaire.

## Introduction

Current thinking about bullying reflects a growing understanding of the concept as a social and cultural issue associated with long-term serious physical and psychological consequences for victims (the *bullied*), aggressors (the *bullies*), and those kids and teenagers simultaneously oscillating between these two roles [1, 2]. Besides empirical research experiences already performed in several countries, data prevalence is wide and variated, but sketches a field with clear and immediate relevance for both present and future welfare-related outcomes of children and young people [3].

The existing data indicate that, globally, almost 1 out of each 3 school students (32%) have been bullied once or more during the last month, while 1 out of each 13 (7.3%) of them might had been bullied on 6 or more days over the same period [4]. Likewise, Juvonen & Graham [2] do not only estimate that approximately 20%– 25% of kids and teenagers might be whether perpetrated and/ or suffered from bullying, but also highlight the social stigma that it represents for youth, as it may contribute to sharpen gender and social disparities [5].

Although the prevalence is similar across genders, the available statistics show how, same as for other aggression-related situations, boys tend to be more involved in fights or physical attacks, whereas girls act more indirectly or relationally [4, 6, 7]. The worldwide prevalence of bullying among girls is estimated to oscillate between [28.2%– 30.4%], while in boys it may range between [30.5%– 34.8%] (UNESCO, 2019). About the modalities, a study identified a prevalence of 35% for traditional bullying (both perpetration and victimization roles) and 15% for cyberbullying involvement [8, 9]. Consistent with this data, other studies rated peer violence across 11 European countries and revealed a similar pattern: 20% of youth between 8 to 18 years reported being bullied, 43.1% of boys and 40.1% of girls remained frequently bullied during secondary school [10]. The youth peer abuse can be persistent across time and across settings [11]; and lastly, victimization was more prevalent among boys and tended to slightly decline with age, especially when interventions, environmental changes (*e*.*g*., school transfer, enter the university) or great variations on social dynamics take place [5, 12, 13].

### “Bullying”: Some key theoretical roots and empirical hints to understand the problem

Conceptually speaking, the definition of *bullying* includes three criteria: intention of harm, repetitiveness, and power imbalance [14]. This systematic abuse of power could culminate in victimization, the repeated occurrence of abuse between peers -from the same age group- where an imbalance of power makes it difficult for the victims to defend themselves [12, 15, 16]. To this effect, bullying-related victimization can be considered as a major stressor within the sphere of peer interactions, whose relevance for social development and academic performance is has been endorsed by previous studies [17, 18].

Moreover, *traditional* bullying victimization has shown connections with a range of negative psychological outcomes including psychosomatic problems, being also associated with an increased likelihood of Deliberated Self-Harm (DSH) [19–21]. In consequence, psychological symptomatology associated to bullying posits decreased belonging in peer networks, increased perceived burden and worry, low self-esteem, and internalizing behaviors which may explain the association between bullying victimization and DSH. Similarly, studies like Kim et al. [22] and Cook et al. [23] described peer rejection and other mental health problems as possible mechanisms by which bullying victimization and DSH may be related. Hong, et. al. [24] suggested that with increasing age, youth get systematically exposed to victimization for longer periods of time; therefore, older teenagers (*i*.*e*., ages 16–18) get involved in longer and more pervasive victimization experiences than youth in early- and mid-adolescence.

As a consequence of these bullying-related victimizations, teenagers commonly report to experience a substantial number of difficulties, including internalizing problems, psychological distress, low life satisfaction relational problems (*i*.*e*., links and relationships with peers, family, school and community members get impaired), and considerable impairments in terms of academic performance [12, 13]. Armitage et al. [3] described victimization during adolescence as a significant risk factor for not only the onset of depression but also poor wellbeing in adulthood, setting a rate over 15% of victims of frequent bullying had a diagnosis of depression at age 18. Not surprisingly, a career as a bully in school predicts increased risks of violence and abuse in later life [25].

Concerning the large literature addressed to the topic, several interesting longitudinal and cross-sectional studies revealed strong relationships between bullying and physical, mental and social health outcomes in victims, bullies and bully-victims [26–32]. Evidence which is also synthetized and supported by numerous meta-analyses [13, 33–39]. As it is clear from this brief summary, bullying among young peers as a worldwide phenomenon is a complex [14] and widespread [4] public health issue that affects mental health and well-being of children of all ages and adolescents [1, 3, 12].

### School-based preventative interventions to reduce bullying: The case of the Valencian Community

Unfortunately, interventive and preventive actions for eradicating bullying from schools have not been always supported by systematic research experiences and protocols. This has enhanced various gaps, such as a limited number of tools to assess bullying at school, what might be useful to develop further empirical-based interventions with a good contextual knowledge [6]. However, the paradigm is changing, and more systematic actions are starting to rise worldwide [40–42]. Currently, one of the most widely adopted approach is the Olweus Bullying Prevention Program (OBPP; [15]), a comprehensive and system-wide program designed to reduce peer violence and achieve better relations among school-aged children and adolescents [43]. Their findings, based on implementation of the intervention in 70 Norwegian schools, reveal positive long-term school-level effects of the program [14, 44].

Another case worth to remark comes from Finland. The so-called “KiVa”, an antibullying program developed by the University of Turku, has been endorsed as a highly effective program relying on enhancing bystanders’ awareness, empathy and self-efficacy to support victimized peers, instead of reinforcing the bullies’ behavior [45]. Indeed, established bullying prevention programs, like OBPP or KiVa, are shown to be effective in reducing bullying through the use of universal, school-wide, and classroom approaches (Hemphill & Smith, 2010). Concretely, several school-based interventions including anti-bullying policies have shown effectiveness in reducing the violence rates by about 20% [46–50].

But by necessity, these anti-bullying programs should also target those who are part of the local, community and national levels to create a multifaceted program that is a permanent component of the school ecology, and not just a temporary solution to bullying behavior. Thus, whole school approaches and bullying prevention-based interventions addressed from a social-ecological perspective have also been shown to significantly reduce bullying behaviors of adolescents [51]. Based on this ecological approach proposed, several protective factors related to the analysis of peer violence have been studied [52–54].

As expected, not only the school, peer relations and individual factors (self-esteem, empathy, and academic performance) are conditional elements for bullying, but the community and family are decisive social spheres where promotion and prevention must be taken into consideration [50, 55]. Given that, interventions for bullying problems are usually focused broadly on systemic change rather than limiting the focus to controlling a child with aggressive behaviour problems or fortifying a child who is victimized.

Another interesting intervention example is provided by Ortega et al. [54], who developed interventions for schools and communities in Spain based on learning together with solidarity, fraternity, cooperation, harmony and a desire for mutual understanding. Their first project (SAVE, Seville Anti-Violence in School) came up with a global process of intervention liked to research and was applied also in secondary education with the aim to involve staff in the slow process of making decisions about the problem, to help teachers distinguish and pay attention to bullying phenomena. Diaz-Caneja et al., [56] performed the web-enabled LINKlusive intervention program at some secondary schools from Madrid (Spain). They also involved all educative agents and focused the 12-week intervention component in identifying bullying situations and by pursuing the student program on the promotion of respect for diversity.

### Some gaps and shortcomings for assessing bullying in the school context

Although some advances on the matter have been developed during the last years, the evaluation of the ecological anti-bullying programs remains often complex due to the variety of methods, the multiple components targeted at different levels of influence (individual students, parents, classrooms, whole schools) and because the evaluation in combination, rather than separately, is recommended [46, 57]. Although very short in number, some similar intervention strategies have been tackled by the Valencian government, after an institutional report [58] warned educational agencies about the need to detect, create indicators and develop criteria that make possible to evaluate bullying at Valencian schools.

The *Educative Inclusion Department* created by the *Regional Ministry of Education*, *Culture and Sports* [*Conselleria d’Educació*, *Cultura i Esport*] in 2015, oriented new guidelines for the early bullying detection and a holistic educative intervention, but also started to undertake a comprehensive process of peer-violence registration. PREVI plan has been established in order to prevent violence and promote a healthy school climate of convivence [59], working through the leadership of 3 emergent specific unities called UAIs (Action-Intervention Units). These protocols for action are designed to intervene in cases of school violence, collaboratively with educative commissioners, local institutions and, at the same time, coordinate and advise the management teams at the educational community. The latest institutional report available [60], corresponding to the academic year 2018, shed light on different interventions implemented by the UAIs at Valencian schools: the 17.7% of the global procedures were related to traditional bullying and cyberbullying notifications, with an increment of 6 percentage points in comparison with the previous school year (2017).

In peer-violence situations, students are assisted by CIC (school living and equality coordinators), professors who are -among other pedagogical aspects- specially trained in bullying prevention and intervention; each secondary school has permanently its own CIC. Parallelly to the assistance, PREVI plan is articulated in several preventive protocols. These programs are grounded in affecting educative curriculums and advocating transversal policies in order to cope with the peer-violence at schools.

Web-enabled resources, like REICO platform [61], aim to offer pedagogical facilities for teachers and families to work with students under this approach to all educative levels. In terms of prevalence in Spain data turns to be clear with the findings of the HBSC Survey [62]: victimization is mainly exerted at 11 to 12 years (15.6%), whereas at 17 to 18 years, it is at 7.4%. In the case of perpetration, this phenomenon seems to be increased in Secondary Education (13 to 15 years), decreasing again in the following years. However, and even though life changes such as the completion of school studies, dropping out of school or "overcoming" a particular aggression source might help to alleviate victimization events, it does not guarantee a full recovery of its "survivors". In fact, empirical studies observed that both the experience and patterns of victimization can be later reflected in further stages of, *e*.*g*., adult life, where social relationships might sometimes involve a victim, sometimes a persecutor, and often both [63, 64]. In practical terms, developing new knowledge and therapeutic programs on this matter would increase the chance of finding adequate insights and solutions to face this challenging panorama [64, 65].

### Searching for “valid” instruments to bullying-school detection

The wide variation in prevalence rates of bullying across studies can be partly attributed to operational differences in the bullying construct, which also affect how it is measured [57]. In addition, definitions of bullying-like phenomena show linguistic variation and may be influenced by what is viewed as legitimate from a cultural point of view. This makes choosing measurement tools a certainly difficult task, given that, for instance, many instruments whether do not use a comprehensive terminology, or target to exclusively address issues related to bullying victimization, omitting the assessment of its potential coexistence with intimidation attitudes and/or behaviors.

Within the multidimensionality of the school climate–and the fact that there is no international consensus on school climate measurement indicators [66], the difficulty to choose the suitable questionnaire becomes a challenge. Bullying assessment used to include questionnaires focused on the study and evaluation of bullying behaviors and their associations related to school climate [67, 68]. Nevertheless, some of these mentioned studies developing peer violence detection instruments could have limitations and psychometric problems. For instance, linked to the response format, ambiguous behaviors references, incorrect formulation of the items, as well as problems of standardization derived from the samples used have been detected, which makes it difficult to correctly calculate their validity and reliability [68, 69].

Based on some questionnaires used in other bullying prevention programs *(e*.*g*., KiVa) we found scales investigating specific behaviors related to the school-environment by measuring bullying “at school” or by “students” and has been used in Spanish students [70]. For instance, CIMEI [6, 54], a 32-item scale was used to measure the incidence of bullying in five schools of secondary education located in Valladolid, Spain [65].

Also, attitudes and beliefs towards violence have been also explored with the CAHV-25 and CONVIVE tools, obtaining a revised version of the tool, testing primary and secondary students in Murcia, Spain. It was also applied in multiple contexts and translated in other languages, but the original consistency found was not very high, and the used of reverse items could have led to confusion, responded bias and lacked robust indicators in factor analysis [68, 71].

Certainly, since the first construct used by Olweus [15] exploring peer relations among children and adolescents, researchers are still searching for a suitable tool to measure peer-violence at schools. Given that the CIE-A: (*i*) proposes measuring “both sides of the coin” (*bullied* and *bully*-related factors) in a multidimensional perspective; (*ii*) uses a simple and comprehensive everyday language; (*iii*) has shown good psychometric properties in the Latin American context (keeping many common factors and dynamics with Spain); and, (*iv*) given its scoring criteria, could be crossed with other health and well-being indicators, it was a prori considered as a reasonability suitable questionnaire to be tested for bullying measurement in our study.

### Study aim

Bearing in mind the aforementioned considerations, including the concerning state-of-affairs on the matter, the lack of research on the field and the need of counting on psychometrically suitable and valid tools to detect school bullying, the aim of this study was to thoroughly present in detail the psychometric properties and validity issues of the School Bullying Questionnaire (CIE-A), tested in a sample of secondary students across the Valencian Community (Spain).

## Materials and methods

### Participants

This study used the data provided by a regionwide sample of *n* = 810 public secondary school students from 21 different districts of the Valencian Community, with a mean age of *M =* 14.40 (SD = 1.61) years. 99% of participants ranged [12–17], while 1% were aged [18–21]. 47.2% of the study participants were females (*girls*) and the remaining 52.8% were males (*boys*). Although a third (“no-binary”) option was available in the survey, this box was not chosen by any of the participants. Also, all study partakers were currently coursing a grade between first year of mandatory secondary studies (consisting of four years–also known as *ESO*) and the first year of baccalaureate (non-mandatory secondary education). A detailed summary on participants’ basic sociodemographic features is presented in Table 1.

**Table 1:** Sociodemographic characteristics of the study participants.

Variable	Group/value	n	%
**Gender**	Female	382	47.2%
	Male	428	52.8%
**Current grade /school year**	1^st^ Secondary	206	25.4%
	2^nd^ Secondary	212	26.2%
	3^rd^ Secondary	103	12.6%
	4^th^ Secondary	180	22.2%
	1^st^ Baccalaureate	109	13.5%
**Have you repeated a grade in your last 5 years of school?**	Yes	215	26.5%
	No	584	72.1%
**Use of social networks**	Never	37	4.6%
	Rarely	28	3.5%
	Weekly or less	27	3.3%
	Several times a week	71	8.8%
	Once a day	65	8.0%
	Several times a day	568	70.1%
	N/R (prefers not answering)	14	1.7%

### Study design and setting

In this school-based study, we firstly invited educational centers (schools) to partake in the research. After obtaining the permissions required from key stakeholders (*i*.*e*., curricular program coordinators, teachers and parents’ associations), including informed consent forms, students coursing secondary or basic baccalaureate (non-mandatory secondary) grades were invited to fill up the paper-based survey in the classroom, with assistance of the educational staff and one member or collaborator of the research team. Therefore, this was a non-probabilistic convenience sampling technique, it grounded on the accessibility to the population of interest, plus their willingness to participate in the study. An a priori statistical power analysis allowed to establish a minimum sample size of about *n* = 790 subjects assuming a low-to-medium effect size, an alpha (*α*) level = .05 and a power (*ß*) = .80 [72]. At the individual level, the response rate of the study (*i*.*e*., received and fully filled questionnaires) was about 85%, from approximately 950 individuals invited to participate.

To perform this research, whose data was collected during the year 2019, the Ethics Committee of the University of Valencia was consulted, guesstimating its compliance with the current relevant ethical principles and the Declaration of Helsinki (IRB H01535548125595), and waiving the need for consent from parents or guardians of the minors included in the study, in consideration of the fact that educational centers provided their authorization to collect the data after assessing the research protocol and data protection issues. All participants were initially informed about the importance of answering honestly to all the form, as well as about the non-existence of wrong or right answers.

### Description of the questionnaire

For this study, we used a paper questionnaire in Spanish language, composed of three core sections, divided as follows:

The first part of the survey inquired about sociodemographic data of participants. Information about age (scalar), gender (nominal; male, female, non-binary), current school grade/year (treated as an ordinal variable) and other basic features related to their educational environment, such as the fact of having failed one of the previous academic grades (nominal/dichotomic) [13].

In the second part of the instrument, it was presented the *School Bullying Questionnaire*, or CIE-A for its acronym in Spanish [*Cuestionario de Intimidación Escolar*], originally created by Cuevas [64] and validated in Spanish speaking (Colombian) school students by Moratto, Cárdenas & Berbesí [73]. This 3-point frequency-based Likert scale consists of 36 items ranging from 0 = *I never experience this*, to 2 = *I experience this very often*, theoretically distributed in three factors or dimensions:

*Victimization (F1)*: Bullying victimization situations (*e*.*g*., physical, verbal and social coercion) commonly characterizing the phenomenon in the school environment (example item: “*Somebody threatens me to do things I don’t want to*”). *Symptomatology (F2)*: Psychological and behavioral signs compatible with common reactions to bullying situations, *e*.*g*., anxiety, depression, post-traumatic stress and effects on self-esteem, assessed in a general and non-clinical approach (example item: “*When I come to school I feel fear or anguish*”). *Intimidation (F3)*: Performance of bullying-related behaviors towards other students (*i*.*e*., peers) by the respondents (example item: “*I make fun of some of my peers*”). The contents of the full set of items composing the questionnaire can be accessed in the appendix provided by Moratto et al. [73].

Finally, it was appended a supplementary section including: (*i*) the Goldberg’s General Health Questionnaire (GHQ) in its short version of 12 items (α = .74), a 4-point Likert scale which provides a unifactorial psychological distress measure feasible to be applied in populations of adolescents [74, 75]; and (*ii*) the Diener’s Satisfaction with Life Scale (SWLS), a 7-point Likert scale composed of 5 items (α = .82) assessing individuals’ global life satisfaction as a single factor [76]. The core purpose of measuring such constructs was to assess the concurrent validity of the CIE-A, as it will be detailed in the next section of the paper.

### Data processing (statistical analysis)

Initially, a careful data curation was performed. As the number of incomplete or blank questionnaires (<2%) was considerably low, the sample size (*i*.*e*., fully answered questionnaires) and the statistical power remained large enough, we used listwise deletion to discard incomplete cases, instead of using imputation methods that may lead to inconsistent bias [77].

Once the parsimoniousness of the dimensional assumptions of the questionnaire was endorsed through the EFA analyses, suggesting a fair adjustment of the CIE-A to its items and a 3-factor composition for the scale (3 of the eigenvalues were <1.0), the measurement model was built up by means a rigorous competitive CFA (confirmatory factor analysis), aimed at testing the factorial arrangement of the CIE-A in the light of various possible dimensional compositions, following the SEM (Structural Equation Modeling) paradigm. It is worth mentioning that CFA involves numerous benefits regarding the management of ordinal and non-normally distributed variables [78]. In addition, one important advantage of CFA is the chance of deciding which proposed model has the most suitable and parsimonious fit, thus allowing the assessment of several models under diverse theoretical assumptions. IBM SPSS AMOS for Macintosh (Version 26.0; IBM Corp., Armonk, NY) was employed for building up these models.

As recommended in previous studies, various estimators and coefficients from different types were used to evaluate the model fit (see Useche et al. [79] for further information). These estimators were: Chi-square (*χ*^*2*^); Root Mean Square Error of Approximation (RMSEA); Normed Fit Index (NFI), Tucket-Lewis Index (TLI) Confirmatory Fit Index (CFI). Goodness-of-fit cut-off points were established as: NFI/TLI/CFI indexes > .900, and a RMSEA < .080 suggest an adequate model fit.

Moreover, the appropriateness of the model was assessed as well, employing the strength and coherence of the estimates, added to the absence of great/redundant modification indexes. The internal consistency (or reliability) of the questionnaire and its items was assessed through three different indexes: (*i*) Cronbach’s Alpha (α); (*ii*) McDonald’s omega (ω), having the advantage of taking into account the strength of association between items and factors and item-specific measurement errors, providing more realistic estimates on scale reliability [80]; and (*iii*) Composite Reliability Index (CRI) an additional coefficient ranging from 0 (zero consistency) to 1 (full consistency), mathematically based on the factor loadings and residuals seen in the results of SEM-based confirmatory analyses (CFAs) [81]. It is worth mentioning that, as this was a cross-sectional (one-measure) study, test-retest reliability of CIE-A could not be assessed. As additional reliability measures, both ω and CFI also contribute to overcome the conventional shortcomings of Cronbach’s α_s_ if used as a single indicator to test scale reliability, greatly dependent on fixed loadings for its calculation.

As the assumption of multivariate normality could not be met with the present data, that was preliminary ordinal, and it can to (*e*.*g*.) lead to inflate *X*^2^ (Chi-square) values, and/or to underestimate standard errors, enhancing potentially incorrect inferences when testing model parameters [82], the model was bootstrapped through a Monte Carlo (parametric) procedure. Bootstrap estimation is a re-sampling technique by which multiple subsamples of an identical size are randomly used to test a model, favoring that (*e*.*g*.) the results of the estimates may be bias-corrected, do not present problems of normality, and type I errors (*false positives*) in regression paths can be avoided, and constitutes a reasonable alternative to other estimation methods such as Satorra-Bentler or Weighted Least Square Mean and Variance adjusted (WLSMV), that cannot be performed with AMOS software.

The concurrent validity (coherence of the relationship between the studied constructs and theoretically associated variables) of the CIE-A was assessed by means of three Criterion Variable (CVs) whose relationship with bullying is suggested by literature (*i*.*e*., psychological distress–CV^1^, satisfaction with life–CV^2^, and negative school outcomes–CV^3^). With the aim of testing the directional coherence and significance of the associations among them and each CIE-A factor, bivariate (Spearman’s *rho*) correlations were used. These non-parametric correlations are preferrable over Pearson’s *r* correlations if the data is non-normally distributed, and/or its nature is ordinal [83], as it was the case of this study.

Finally, a gender-based comparative analysis on the dimensional scores of the CIE-A was carried out through Welch’s comparative analyses, a Student’s t-based non-parametric statistical test entailing a considerable set of advantages over parametric tests such as ANOVA, especially if variances are predominantly imbalanced and/or the compared groups’ sizes are disproportionate. IBM SPSS Statistics for Macintosh (Version 26.0; IBM Corp., Armonk, NY) was used to perform descriptive and comparative tests of this study.

## Results

### Structural models

With the aim of understanding the factorial structure of the Spanish version of the *School Bullying Questionnaire* (CIE-A), two competitive CFAs were performed. Firstly, we tested the original structure composed of three factors, and secondly, a possible bifactorial structure, in order to make fit comparisons and determining the best possible theoretical structure for the scale. The model fit for the bifactorial solution was considerably inadequate: *χ*^2^_(593)_ = 3378.183, *p<* .001; RMSEA = .076 with 90% CI of .074-.079; CFI = .769; NFI = .734; IFI = .770. On the other hand, the baseline three-factor model showed better fit indexes, with: *χ*^2^(591) = 2205.321, *p<* .001; RMSEA = .058 with 90% CI of .056-.061; CFI = .866; NFI = .826; IFI = .867.

A close inspection of this unconstrained three-factor model allowed us to identify a reduced set of very large modification indexes that pointed out a relevant relationship between some items. The new simplified model fitted the data reasonably well, presenting the following fit indices: *χ*^2^_(551)_ = 1254.045, *p<* .001; RMSEA = .040 with 90% CI of .037-.043; CFI = .941; NFI = .901; IFI = .942, as presented in Table 2.

**Table 2:** Competitive CFA–goodness-of-fit indices obtained for the structural models.

Model	*X* ^2^	*p* ^a^	RMSEA^b^	90% CI^c^		CFI^d^	NFI^e^	IFI^f^
				Lower	Upper			
1. Bifactorial solution	1662.237	< .001	.078	.075	.082	.727	.695	.701
2. Three-factor solution	1447.489	< .001	.064	.061	.067	.866	.826	.867
4. Three-factor adjusted solution (retained)*	1254.002	< .001	.040	.037	.043	.941	.901	.942

Notes for the table

^a^ *p*-value

^b^ Root Mean Square Error of Approximation

^c^ Confidence Interval for RMSEA at the level 90%

^d^ Confirmatory Fit Index

^e^ Normed Fit Index

^f^ Incremental Fit Index.

**Fig 1:**
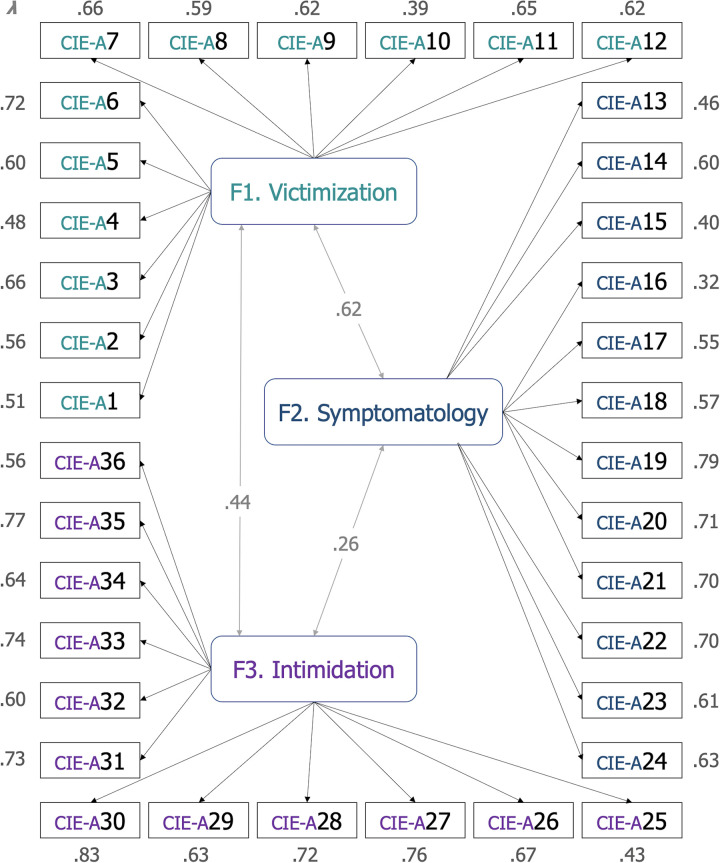
CIE-A structure. Standardized parameter estimates and factor correlations. *Notes*: All standardized estimates were *p <* .001; the numbers within squares represent the original numbers of the items in the CIE-A (as shown in Table 3).

It is relevant to remark that when this model fit is compared to a bifactorial solution with the same set of items, the final three-factor structure presents a much better fit without the need of deleting questions, bearing in mind both the considerably adequate factor loadings (all *λ* > .40) and the reliability scores obtained in the following analysis (see *3*.*2 Internal consistencies*). Table 3 shows the content, descriptive data (average scores and standard deviations), standardized factor loadings and significance levels of each one of the items composing the CIE-A, as also shown in Fig 1.

**Table 3:** Item descriptive—factorial composition and bootstrapped bias-corrected coefficients of the retained three-factor model for the CIE-A.

Item	Content	Factor	M^a^	SD^b^	λ^c^	S.E.^d^	C.R.^e^	*p* ^f^	Bootstrap bias-corrected values^g^				
									Estimate^h^	S.E.^d^	95% CI^i^		*p* ^j^
CIE1		Factor 1: Victimization	.340	.542	.513	.095	12.549	< .001	1.188	.102	1.016	1.368	.009
CIE2			.140	.381	.558	.065	11.753	< .001	.763	.066	.660	.879	.012
CIE3			.170	.428	.665	.077	13.138	< .001	1.015	.077	.903	1.154	.009
CIE4			.410	.584	.480	.094	10.731	< .001	1.007	.095	.847	1.156	.023
CIE5			.200	.483	.599	.084	12.368	< .001	1.039	.076	.936	1.202	.007
CIE6		CRI^k^ = .979	.360	.582	.721	.111	13.623	< .001	1.506	.108	1.330	1.651	.032
CIE7		α^l^ = .855	.200	.475	.657	.086	13.045	< .001	1.123	.091	1.004	1.298	.009
CIE8		ω^m^ = .878	.320	.556	.593	.096	12.369	< .001	1.183	.104	1.043	1.378	.012
CIE9			.170	.463	.627	.082	12.723	< .001	1.043	.083	.925	1.217	.012
CIE10			.630	.731	.389	.111	9.227	< .001	1.020	.111	.813	1.170	.028
CIE11			.110	.373	.651	.066	13.006	< .001	.854	.070	.754	.993	.009
CIE12			.110	.376	.625	.067	12.549	< .001	.842	.075	.731	.984	.011
CIE13		Factor 2: Symptomatology	.480	.665	.465	.063	11.747	< .001	.737	.059	.637	.839	.007
CIE14			.240	.478	.598	.081	11.439	< .001	.923	.079	.801	1.064	.013
CIE15			.480	.608	.404	.088	9.030	< .001	.795	.090	.657	.947	.011
CIE16			.560	.719	.322	.098	7.641	< .001	.749	.095	.622	.939	.008
CIE17			.500	.644	.552	.105	10.949	< .001	1.150	.106	.996	1.344	.014
CIE18		CRI^k^ = .977	.170	.447	.568	.073	11.216	< .001	.818	.081	.695	.968	.010
CIE19		α^l^ = .867	.360	.612	.795	.122	12.960	< .001	1.576	.130	1.392	1.802	.012
CIE20		ω^m^ = .870	.420	.617	.709	.114	12.413	< .001	1.410	.120	1.206	1.584	.014
CIE21			.300	.569	.697	.104	12.303	< .001	1.283	.104	1.110	1.447	.016
CIE22			.460	.633	.701	.116	12.336	< .001	1.430	.116	1.265	1.647	.010
CIE23			.560	.687	.614	.117	11.523	< .001	1.351	.126	1.161	1.594	.009
CIE24			.460	.664	.632	.115	11.747	< .001	1.356	.115	1.193	1.570	.013
CIE25		Factor 3: Intimidation	.140	.389	.426	.064	10.463	< .001	.668	.064	.557	.768	.011
CIE26			.070	.295	.675	.095	12.545	< .001	1.193	.095	1.036	1.345	.009
CIE27			.080	.326	.761	.127	11.757	< .001	1.494	.128	1.274	1.698	.012
CIE28			.050	.258	.724	.097	11.606	< .001	1.131	.092	.978	1.295	.009
CIE29			.100	.350	.645	.122	11.162	< .001	1.363	.114	1.142	1.544	.019
CIE30		CRI^k^ = .901	.070	.308	.829	.131	11.757	< .001	1.540	.123	1.325	1.749	.008
CIE31		α^l^ = .901	.100	.340	.727	.128	11.662	< .001	1.488	.118	1.297	1.688	.007
CIE32		ω^m^ = .903	.230	.462	.599	.140	11.956	< .001	1.668	.138	1.464	1.918	.009
CIE33			.110	.366	.741	.140	11.668	< .001	1.638	.127	1.469	1.881	.007
CIE34			.060	.288	.641	.100	11.114	< .001	1.111	.095	.940	1.255	.013
CIE35			.090	.341	.771	.134	11.826	< .001	1.585	.134	1.393	1.857	.006
CIE36			.160	.445	.558	.143	10.473	< .001	1.497	.146	1.301	1.795	.009

Notes for the table

^a^ Mean

^b^ Standard Deviation

^c^ Standardized factor loading

^d^ Standard Error

^e^ Critical Ratio

^f^ All *p*-values were lower than .001

^g^ Bootstrapped (bias-corrected) model

^h^ Unstandardized estimates

^i^ Confidence Interval at the level 95% (lower bound–left; upper bound–right)

^j^ All *p*-values in bootstrap were lower than .010

^k^ Composite Reliability Index

^l^ Cronbach’s alpha

^m^ McDonald’s omega.

### Internal consistencies

Regarding internal consistency, all the three factors of the retained model have shown good-to-optimal values, as follows:

Cronbach’s alpha estimates were all above the usual α = .700 criteria, advised in several specialized sources (Morera & Stokes, 2016), which denotes a suitable internal reliability for all scales: .855 for *Victimization* (Factor 1); .867 for *Symptomatology* (Factor 2); and .901 for *Intimidation* (Factor 3).

McDonald’s omega coefficients were all ω > .870, with: .878 for *Victimization* (Factor 1); .870 for *Symptomatology* (Factor 2); and .903 for *Intimidation* (Factor 3).

Moreover, the Composite Reliability Index (CRI) was also assessed, in order to provide a complementary measure to the two previously described measures, showing highly adequate reliabilities for all the three latent constructs addressed by the scale. CRI for F1 (*Victimization*) was .979. The CRI for F2 (*Symptomatology*) was .977. Finally, CRI for F3 (*Intimidation*) was .901.

Apart from these coefficients, Table 3 also presents in detail the content, descriptive data (arithmetic means and deviations), standardized factor loadings or “lambda” coefficients (*λ*s) and significance levels (all *p* < .001) of each one of the items composing the CIE-A, being all factor loading coefficients in the retained model large, positive and significant at their correspondent dimensions.

### Factor correlations and concurrent validity

Overall, the three dimensions of the CIE-A kept adequate coherent and significant associations, both (*i*) among pairs of them, with all *rho* bivariate correlations having a positive directionality and remaining statistically significant and with a relatively great magnitude, and (*ii*) with criterion variables, that were previously endorsed by literature as potentially (and coherently) associated to bullying. This is: CV1 –Psychological distress, CV2 –Satisfaction with life, and CV3 –Negative school outcomes (*i*.*e*., having failed/repeated at least one school year, as an indicative). The whole set of correlations found between the CIE-A main components (factors) and the criterion variables chosen can be seen in Table 4.

**Table 4:** Concurrent validity (bivariate correlations) between CIE-A factors and theoretically related Criterion Variables (CVs).

Factor		Statistic	F2	F3	CV1	CV2	CV3
**F1**	Victimization	*rho*	.684**	.799**	.324**	-.292**	.129**
		Sig.	*<* .*001*	*<* .*001*	*<* .*001*	*<* .*001*	*<* .*001*
**F2**	Symptomatology	*rho*	1	.495**	.550**	-.410**	.130**
		Sig.	--	*<* .*001*	*<* .*001*	*<* .*001*	*<* .*001*
**F3**	Intimidation	*rho*		1	.175**	-.220**	.131**
		Sig.		--	*<* .*001*	*<* .*001*	*<* .*001*
**CV1**	Psychological Distress	*rho*			1	-.513**	.088*
		Sig.			--	*<* .*001*	*<* .*050*
**CV2**	Satisfaction with Life	*rho*				1	-.121**
		Sig.				--	*<* .*001*
**CV3**	Negative school outcomes ^a^	*rho*					1
		Sig.					--

Notes for the table

^a^ Dummy variable; success = having failed at least one of the last five academic years

** Correlation is significant at the .001 level (2-tailed)

* Correlation is significant at the .050 level (2-tailed).

Some correlations worth noting are the significant associations between factors F1 (Victimization), F2 (Symptomatology) and F3 (Intimidation), and the self-reported scores of Psychological Distress (CV^1^ [74, 75]), that are in all cases positive and significant at the level < .001. On the other hand, greater scores on all three factors measured through the CIE-A were also negatively correlated with the Satisfaction with Life index (CV^2^ [76]; all *p*-values < .001). Finally, the CV^3^ (Negative school outcomes [13]) has been positively correlated with all the CIE-A factors or subscales. In summary, all the correlations were significant and had coherent directions with the hypothesizable in the light of the available literature, thus endorsing the concurrent validity of the instrument and all its three factors.

### Gender-based differences

With the aim of testing the existence of potential differences in the scores measured by the three scales of the CIE-A, and thus endorsing its discriminant ability, the scores provided by students from both genders (*i*.*e*., females and males, as there were no subjects labelling themselves as no-binary) were compared through Welch’s robust tests, that being non-parametric and overcoming the non-normality issues of the responses distribution result more convenient that Analysis of Variance (ANOVA).

Also, Welch’s t-based tests offer certain advantages over similar non-parametric procedures available, as a result of their improved statistical robustness. The results of the gender-based comparisons, including standard deviations, errors and confidence intervals, are displayed in Table 5.

**Table 5:** Descriptive data, confidence intervals and Welch’s robust mean comparisons. Categorical factor: Gender.

Factor	Category	*n*	Sum	M	SD^a^	SE^b^	95% CI^c^		Welch			
							Lower	Upper	Statistic^d^	df1	df2	Sig.^e^
**F1: Victimization**	Female	428	2.602	.217	3.388	.164	2.280	2.924	19.220	1	740.238	< .001
	Male	382	3.770	.315	4.110	.210	3.357	4.184				
	Total	810	3.153	.262	3.789	.133	2.892	3.414				
**F2: Symptomatology**	Female	428	5.756	.480	5.159	.249	5.266	6.246	23.917	1	792.154	< .001
	Male	382	4.181	.348	3.987	.204	3.779	4.582				
	Total	810	5.013	.418	4.707	.165	4.688	5.338				
**F3: Intimidation**	Female	428	.585	.049	1.812	.088	.413	.757	46.263	1	541.455	< .001
	Male	382	1.996	.167	3.677	.188	1.627	2.366				
	Total	810	1.251	.104	2.932	.103	1.048	1.453				

Notes for the table

^a^ Standard Deviation

^b^ Standard Error

^c^ Confidence Interval at 95%

^d^ Asymptotically distributed (*F*)

^e^ p-value obtained for Welch’s Robust Tests of Equality of Means.

In comparative terms, it was found that all the three dimensions measured by the School Bullying Questionnaire (CIE-A) present gender-based differences, as follows: Male secondary students report a significantly greater mean score for the factors F1 (Victimization; Welch’s *t* = 19.220; *p* < .001) and F3 (Intimidation; Welch’s *t* = 46.263; *p* < .001). Said differently, male individuals report to experience bullying victimization situations more frequently than their female counterparts. Nevertheless, boys were also those gender-based group reporting to perform bullying-related behaviors over their peers with a greater frequency, if compared with girls.

**Fig 2:**
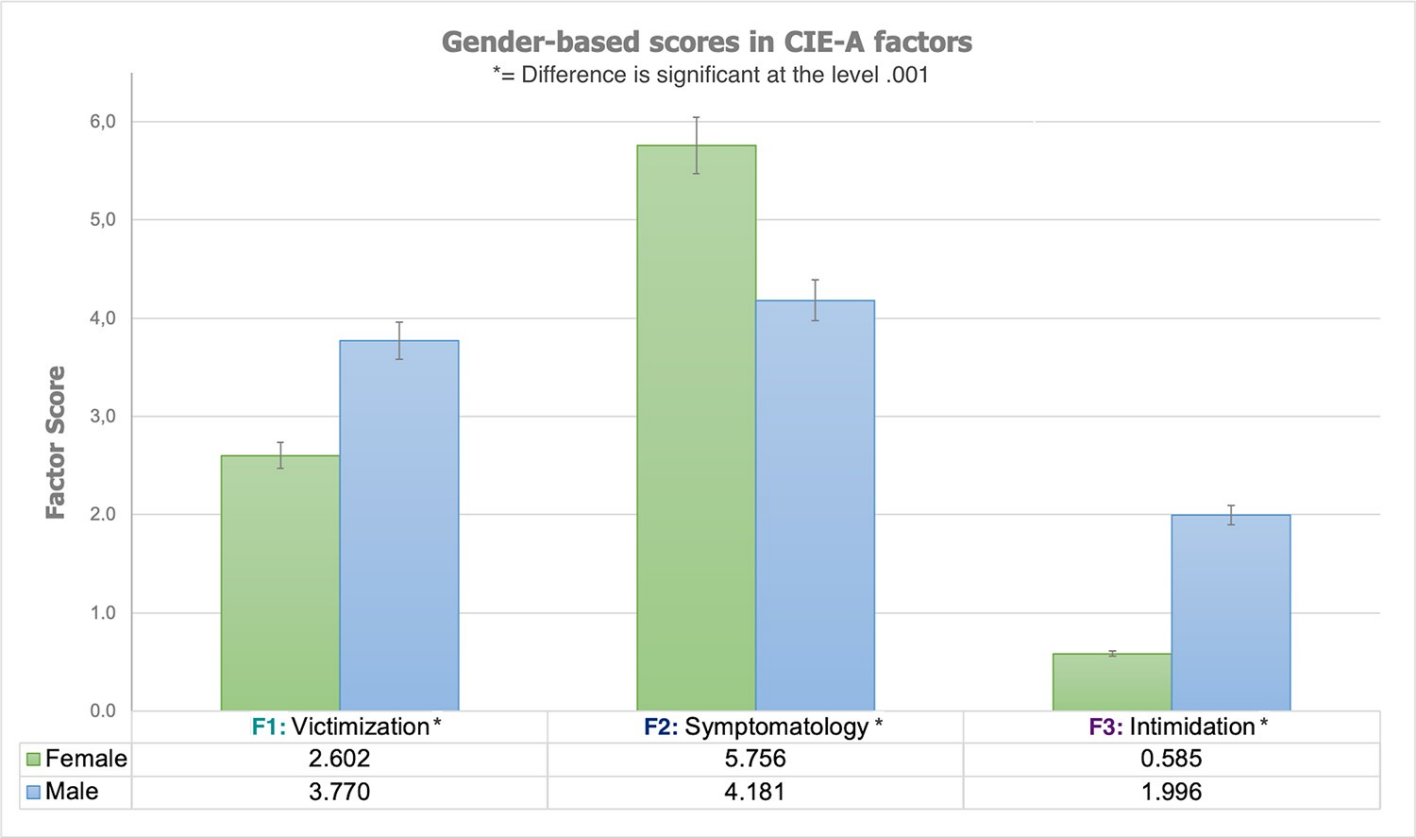
Gender differences. Gender-based score comparisons for the three CIE-A dimensions. Compared values represent aggregate scale scores.

On the other hand, and although they report suffering bullying-related expressions with a lesser frequency, female participants where those having significantly greater mean scores on the F2 (Symptomatology; Welch’s *t* = 23.917; *p* < .001), *i*.*e*., tending to show symptoms potentially understandable as bullying outcomes with a greater frequency than their male peers. The descriptive outcomes and significant differences found through the robust comparative tests are graphically presented in Fig 2.

## Discussion

The core aim of this research was to thoroughly present in detail the psychometric properties and validity insights on the School Bullying Questionnaire (CIE-A), tested across one of the most populated regions of Spain, *i*.*e*., the Valencian Community. Overall, the results of this study support the idea that the tested 36-item version of the CIE-A has a clear dimensional three-factor structure, systematically testing and endorsing its psychometric value and reliability for measuring bullying-related features among secondary students.

### Psychometric strengths and key indexes of the CIE-A

Psychometrically speaking, the three estimated coefficients (all Cronbach’s alphas > .850, McDonald’s omegas > .870, and CRIs > .900) gave good account of a reasonably good set of insights of the psychometrical robustness of the test in terms of internal reliability and consistency. This is coherent to the good Cronbach’s indexes (ranging between α = [.83 - .89]) found by Moratto et al. [73] in their validation of the 36-item CIE-A among Colombian teenagers.

Also, and regarding its structural suitability, all the CIE-A items had a very acceptable factorial weight (all λ > .400), and the questionnaire was satisfactorily adjusted to a parsimonious structure consisting of a three-factorial latent variable model, with the following dimensions included: *Victimization (F1)*, *Symptomatology (F2*); and *Intimidation (F3)*. These labels respond, in the first place, to the content of the items with greater factorial weight for each dimension and, secondly, to the shared theoretical background of the CIE-A regarding other previous studies addressing bullying dynamics and its most commonly related definitions, factors, dynamics and expressions [12, 15, 84].

This psychometric finding (*i*.*e*., the three-dimensionality plausibleness of the CIE-A questionnaire), acquires theoretical significance as it supports the capability of the questionnaire to differentiate between them from the exploratory to the confirmatory phases of the structural analysis. This distinction between bullying-related suffered (F1 and F2) and exerted (F3) victimization becomes crucial when considering that the evidence shows how reproducing similar comportments towards others is a relatively frequent reaction among teenager students having been victimized by bullies, as well as can be understood as a potential method for adaptation to social dynamics at school [1, 9, 34].

### Gender differences and gaps in the CIE-A bullying measurement

Another key analysis presented in this paper was the mean comparison of the three CIE-A factors according to students’ gender, having shown significant mean differences in all the three factors measured by the questionnaire. In this regard, there are two issues worth of discussing: first of all, that these differences were coherent with the current literature on the gender-based differences in terms of these three factors [36, 73], overall showing how male school students tend to be more likely to both suffer and to exert bullying-related behaviors, while females are those commonly reporting greater signs of distress and psychosocial affectation as a consequence of bullying situations, also having a greater stress perception relative to being bullied [6, 24, 34, 84].

Secondly, and still in need of further applications and analyses, these factor-based results and the significance of their mean score differences constitute an inkling of the questionnaire capability to differentiate respondents by gender [7, 10], keeping an adequate coherence with the theoretical roots supporting the instrument approach [84–86], making it possible to obtain a relatively short and accurate self-report-based measurement of its three constructs among teenagers.

Finally, and to the best of our knowledge, this is the first large scale empirical research measuring bullying-related factors among secondary school students in the Valencian Community through the CIE-A. However, previous interventions–whose directions for further research and planning are still worth of consideration–have remarked the need of counting on further research aimed at improving the understanding of dynamics and features of school bullying in the region [6, 48]. Therefore, and given the good set of psychometric properties, validity and reliability indexes of the questionnaire, the CIE-A can be considered as a potentially useful tool to study this threatful issue, and to support evidence-based interventions and policies on the matter.

## Conclusion

Finally, the results of this study suggest that the 36-item version of the School Bullying Questionnaire (CIE-A) represents a suitable tool for assessing bullying in a three-factorial approach among secondary students, offering optimal psychometric properties, validity and reliability insights, and the potentiality of being applied in the school environment.

Therefore, this self-report scale can be suggested as a useful, reliable and valid tool for addressing bullying from a psychometric perspective in educational, psychological, legal and multidisciplinary sciences.

Moreover, practical actions and policies aimed at improving the school coexistence and the well-being of secondary school students, focused on targeting potential *bullied* and/or *bully* profiles or seeking to assess its demographic and psychosocial correlates, might get benefited from this measurement tool.

### Limitations of the study and further research

Although this study covered a relatively extensive research sample, a priori statistically representative of the study population on a regional basis (*i*.*e*., secondary school students of the Valencian Community, Spain) and the statistical parameters required for each test support the suitability and reliability of their outcomes, there are some key limitations and shortcomings that should be, at least, briefly acknowledged, in order to give our readers a transparent overview and enough context to interpret the outcomes of this study. Firstly, this was a cross-sectional design, thus limiting us in terms of temporality; therefore, neither the development of the factors measured nor the CIE-A test-retest reliability indexes can be estimated through the data retrieved [87]. Secondly, and same as most of in-school applied researches on bullying addressing big samples, this study was based on self-reported information, that is prone to be potentially biased by the so-called Common method Biases (CBMs) or Common Method Variance (CMV), ranging from deliberately showing social desirability in their responses to reflect–even undeliberate ways–gender-based roles in social settings [88, 89].

Regarding further issues to consider, and as this study was carried out within the school environment, it would be interesting to: (*i*) further apply this research tool across other regions and/or Spanish-speaking countries (given that the questionnaire used a very generic and cross-culturally understandable terminology); (*ii*) test the potential differences explainable by socio-demographic variables different to gender (*e*.*g*., type of school, income level, family and microsocial features); (*iii*) assess potential changes or “mutations” of bullying (*e*.*g*., to what extent has it shifted to *cyber-bullying*) as a result of the growing of social distancing and remote schooling; and (*iv*) address other stakeholders’ perspectives, potentially involving complementary data sources or *proxies* (*e*.*g*., teachers, coordinators and parents), in order to qualitatively enrich the outcomes of the quantitative measures provided by the CIE-A questionnaire.

## Supporting information

S1 File Raw data. Raw data is available in the file (database) attached to the electronic version of this manuscript.
(SAV)
